# Supplementary material for: Nanoplastics and Arsenic Co-Exposures Exacerbate Oncogenic Biomarkers under an In Vitro Long-Term Exposure Scenario
Source: Int J Mol Sci. 2022 Mar 9;23(6):2958. doi: 10.3390/ijms23062958 (PMC8955425; doi:10.3390/ijms23062958)
Supplement: Supplementary file 1 [file ijms-23-02958-s001.zip › ijms-1604419-supplementary.pdf]

Nanoplastics and arsenic co-exposures exacerbate oncogenic biomarkers under an *in vitro* long-term exposure scenario

Irene Barguilla[1], Josefa Domenech[1], Laura Rubio[2], Ricard Marcos[1,§], Alba Hernández[1,§]

SUPPLEMENTARY MATERIAL

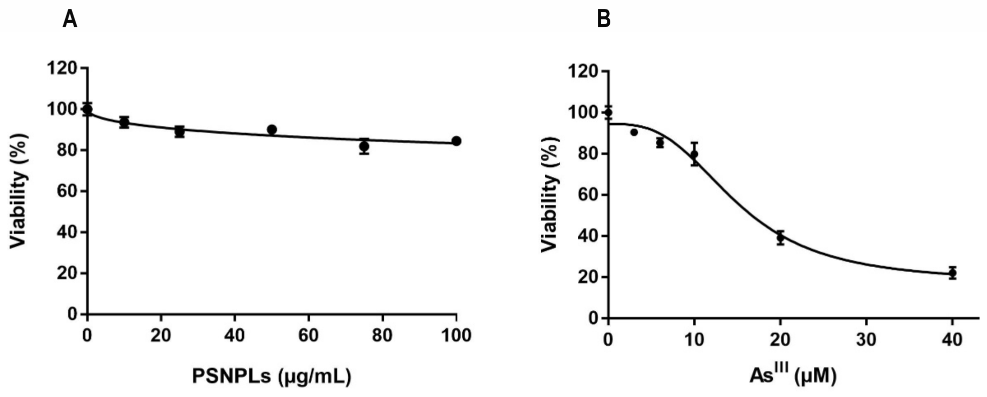

Figure S1. Relative survival of PTP cells after 24 h of exposure to PSNPLs (A) and As^{III} (B). Data are presented as the percentage of counted cells relative to the untreated control ± SEM.
